# Structure-based dynamic arrays in regulatory domains of sodium-calcium exchanger (NCX) isoforms

**DOI:** 10.1038/s41598-017-01102-x

**Published:** 2017-04-20

**Authors:** Moshe Giladi, Su Youn Lee, Yarden Ariely, Yotam Teldan, Rotem Granit, Roi Strulovich, Yoni Haitin, Ka Young Chung, Daniel Khananshvili

**Affiliations:** 1 Department of Physiology and Pharmacology, Tel-Aviv University, Tel-Aviv, 69978 Israel; 2 School of Pharmacy, Sungkyunkwan University, Jangan-gu, Suwon 440-746 South Korea

## Abstract

Mammalian Na^+^/Ca^2+^ exchangers, NCX1 and NCX3, generate splice variants, whereas NCX2 does not. The CBD1 and CBD2 domains form a regulatory tandem (CBD12), where Ca^2+^ binding to CBD1 activates and Ca^2+^ binding to CBD2 (bearing the splicing segment) alleviates the Na^+^-induced inactivation. Here, the NCX2-CBD12, NCX3-CBD12-B, and NCX3-CBD12-AC proteins were analyzed by small-angle X-ray scattering (SAXS) and hydrogen-deuterium exchange mass-spectrometry (HDX-MS) to resolve regulatory variances in the NCX2 and NCX3 variants. SAXS revealed the unified model, according to which the Ca^2+^ binding to CBD12 shifts a dynamic equilibrium without generating new conformational states, and where more rigid conformational states become more populated without any global conformational changes. HDX-MS revealed the differential effects of the B and AC exons on the folding stability of apo CBD1 in NCX3-CBD12, where the dynamic differences become less noticeable in the Ca^2+^-bound state. Therefore, the apo forms predefine incremental changes in backbone dynamics upon Ca^2+^ binding. These observations may account for slower inactivation (caused by slower dissociation of occluded Ca^2+^ from CBD12) in the skeletal vs the brain-expressed NCX2 and NCX3 variants. This may have physiological relevance, since NCX must extrude much higher amounts of Ca^2+^ from the skeletal cell than from the neuron.

## Introduction

The properties of Ca^2+^-binding proteins that participate in Ca^2+^-dependent regulation of distinct cell-signaling pathways are controlled by tissue-specific expression and alternative splicing of closely related genes^1^. The isoform-dependent and alternative splicing-dependent modification of Ca^2+^-binding regulatory domains are especially important for Ca^2+^-transporting proteins operating in excitable tissues^1–3^, since feedback interactions of Ca^2+^ with allosteric regulatory domains dynamically modulate the Ca^2+^-transport rates in accordance with dynamic changes in cellular Ca^2+^ oscillations (e.g., in cardiomyocytes during the action potential)^1, 3–5^. Despite the fundamental importance of this issue, the structure-dynamic determinants governing the isoform-dependent and alternative splicing-dependent modification of regulatiory specificity in Ca^2+^-binding domains remain poorly understood^2, 6^.

**Figure 1:**
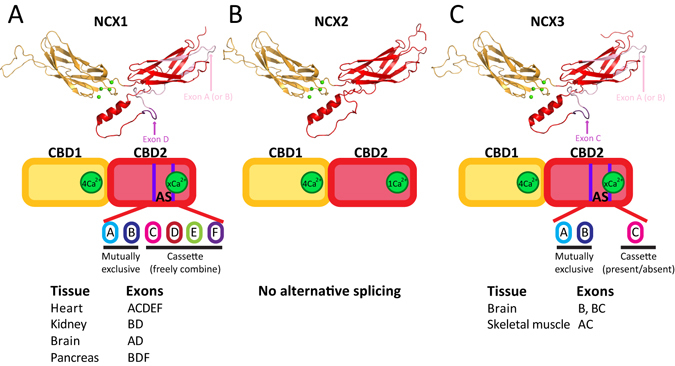
Structure and alternative splicing of NCX-CBD12 isoforms. The models of NCX1-CBD12-AD (brain splice variant) (**A**), NCX2-CBD12 (**B**), and NCX3-CBD12-AC (**C**) are presented as cartoons. CBD1 is in orange and CBD2 is in red. Four Ca^2+^ ions bound to CBD1 are presented as green spheres. The alternative splicing schemes of NCX1 (**A**), NCX2 (**B**), and NCX3 (**C**) are presented below the models.

Na^+^/Ca^2+^ exchanger (NCX) proteins serve as a major route for Ca^2+^ extrusion from cells^3, 4^. In mammals, three gene isoforms (SLC8A1, SLC8A2, and SLC8A3) of NCX proteins (NCX1, NCX2, and NCX3) and their splice variants are expressed in a tissue-specific manner, while exhibiting distinct regulatory phenotypes (Fig. 1)^7–10^. The activity of NCX proteins is “secondarily” modulated by cytosolic Na^+^ and Ca^2+^ ^8–10^. In mammals, an increase in cytosolic [Na^+^] usually inactivates NCX, whereas an increase in cytosolic [Ca^2+^] usually activates NCX^3, 4, 6^. However, the appearance of these regulatory modes as well as their strength and duration vary in an isoform and splice variant-dependent manner, since the regulatory features of a given isoform/splice variant must match the tissue-specific contributions of NCX to the dynamic handling of Ca^2+^ homeostasis in distinct cell-types^7, 9, 11^. For example, in some isoforms and splice variants, an increase in the cytosolic [Ca^2+^] can alleviate Na^+^-dependent inactivation, whereas in others, Ca^2+^ cannot alleviate Na^+^-dependent inactivation, or the regulatory mode of Na^+^-dependent inactivation does not exist at all^7, 9, 11, 12^.

NCX1 is universally distributed, practically in every mammalian cell, although the NCX1 splice variants are selectively expressed in a tissue-specific manner (Fig. 1A)^8, 10^. Alternative splicing of NCX1 results in at least 17 splice variants, where either exon A or exon B (mutually exclusive exons) can be freely combined with cassette exons (C, D, E, and F) (Fig. 1A)^3, 4, 8, 10^. In general, exon-A-containing NCX1 splice variants are mainly expressed in excitable tissues (e.g., cardiomyocytes, neurons), whereas exon-B-containing NCX1 splice variants are mainly expressed in non-excitable tissues (e.g., pancreas, kidney) (Fig. 1A)^3, 4, 8, 10^. NCX2 is predominantly expressed in the brain and spinal cord, but it can also be found in the gastrointestinal tract and kidney tissues. NCX2 does not undergo alternative splicing (Fig. 1B). NCX3 is predominantly expressed in the brain and skeletal muscle, but it can also be found in osseous tissue and the immune system^8, 10, 11, 13^. Alternative splicing of NCX3 results in at least 5 splice variants and involves the expression of either exon A or exon B (mutually exclusive exons) in the presence or absence of an additional exon C (Fig. 1C)^8, 10, 11, 13^. Exon-A-containing NCX3 splice variants are mainly expressed in skeletal muscle, whereas exon-B-containing NCX3 splice variants are mainly expressed in the brain (Fig. 1C)^11^.

Prokaryotic and eukaryotic NCX proteins contain ten transmembrane helices (TM1–10), where the ion-binding pocket selectively binds either one Ca^2+^ or three Na^+^ ions and transports them in separate steps^14–17^. Eukaryotic (but not prokaryotic) NCX proteins contain a large cytosolic loop between TM5 and TM6, mainly composed of two Ca^2+^-binding domains, CBD1 and CBD2^18^. The two CBDs form a head-to-tail attached two-domain tandem (termed CBD12) through a very short interdomain linker (Fig. 1)^18, 19^. The Ca^2+^-dependent activation of NCX1 results from Ca^2+^ binding to CBD1^12^, whereas the Ca^2+^-dependent alleviation of Na^+^-induced inactivation results from Ca^2+^ binding to CBD2^12, 20, 21^. Notably, the Na^+^-dependent inactivation of NCX is due to Na^+^ interaction with the ion-transport domains of NCX and not with CBD12^22, 23^, although Ca^2+^ binding to NCX1-CBD2 somehow relieves the Na^+^-induced inactivation via propagation of an allosteric signal over a large distance from CBD12 to transport domains^6^.

NMR^18^ and X-ray crystallography^20, 24^ studies revealed that CBD1 and CBD2 share a similar structure, exhibiting an immunoglobulin-like β-sandwich structure with seven antiparallel β-strands (Fig. 1). CBD1 contains four Ca^2+^ binding sites, capable of high-affinity Ca^2+^ sensing (K_d_ < 1 µM) in all NCX orthologs, isoforms, and splice variants^6, 21, 24–26^. The Ca^2+^ binding sites of CBD1 are located at its C-terminal tip, near the CBD1-CBD2 interface (Fig. 1)^19, 24^. In contrast, the number of Ca^2+^ binding sites at CBD2 varies from zero to three, depending on the specific ortholog, isoform, and splice variant (Fig. 1)^21, 25, 26^. The affinity for Ca^2+^ binding at CBD2 is rather low, ranging from ~5 to ~200 μM^21, 23, 25, 26^. Notably, alternative splicing of NCX (described in detail above) takes place only at CBD2 and involves the Ca^2+^ binding sites^18, 21^. Therefore, in A-exon-containing NCX1 variants, CBD2 binds two Ca^2+^ ions, whereas in B-exon-containing NCX1 variants, CBD2 does not bind Ca^2+^ ions (Fig. 1A)^21, 25^. Since Ca^2+^ binding to CBD2 alleviates Na^+^-induced inactivation, this effect of Ca^2+^ is only observed in A-exon-containing NCX1 variants^7, 12, 21^. In NCX2, CBD2 binds only one Ca^2+^ ion with very low affinity (Fig. 1B)^21, 26^. Since NCX2 does not exhibit any Na^+^-dependent inactivation, the Ca^2+^-dependent alleviation of Na^+^-induced inactivation is functionally irrelevant with NCX2^9^. In NCX3, A-exon-containing variants do not bind Ca^2+^ ions at CBD2, whereas B-exon-containing variants bind three Ca^2+^ ions at CBD2 (Fig. 1C)^26^. Intriguingly, NCX3-AC (which does not bind Ca^2+^ at CBD2) was shown to exhibit less Na^+^-dependent inactivation compared with NCX3-B (which does bind Ca^2+^ at CBD2) in electrophysiological studies^11^.

Recent studies using diverse biophysical, biochemical, and structural methods have identified synergistic interactions between the CBDs that shape the dynamic range and kinetic features of Ca^2+^-dependent regulation in splice variants of NCX1, thereby providing the insights into the mechanisms underlying CBD interactions in isolated CBD12 and full-size NCX1^18–21, 24–32^. These studies have shown that the isolated preparations of CBD12 largely represent the Ca^2+^ sensitivity and kinetics of Ca^2+^-dependent regulation in their matching of full-size NCX isoforms and splice variants^7, 25, 30^. Therefore, isolated CBD12 serves as an ideal model for investigating the structure-dynamic mechanisms underlying the allosteric regulation specificities in NCX isoforms and splice variants. The interactions between CBD1 and CBD2 in the context of CBD12 results in increased affinity for Ca^2+^ at CBD1 and slow dissociation of an “occluded” Ca^2+^ ion from CBD1^25, 27^. In addition, alternative splicing at CBD2 not only affects the Ca^2+^ binding affinity and capacity at CBD2—it also affects the Ca^2+^ interactions with CBD1, establishing up to 50-fold differences both in the Ca^2+^ binding affinity and Ca^2+^ off-rates in the cardiac (ACDEF), brain (AD), and kidney (BD) splice variants of NCX1^25, 27^. Using ^45^Ca^2+^ equilibrium binding and stopped-flow kinetic assays, we have recently demonstrated that NCX2 and NCX3 also exhibit alternative-splicing dependent synergistic interactions between the CBDs, similarly to NCX1^26^. NCX3-CBD12 splice variants exhibit similar Ca^2+^ affinity at CBD1, but the dissociation rate of the occluded Ca^2+^ ion is ~10-fold slower in skeletal muscle (AC) than in the brain (B) variants^26^. These differences in Ca^2+^ binding affinity and the Ca^2+^ off-rates at the primary allosteric sensor (CBD1) may have physiological relevance for diversifying regulatory responses in full-size NCX variants expressed in a tissue-specific manner^1–3, 6^.

X-ray crystallography revealed that Ca^2+^ binding to CBD1 results in Ca^2+^ occlusion and tethering of CBD1 and CBD2 through the formation of a hydrogen-bonded salt-bridge network at the two-domain interface, yielding a more stable structure^6, 19^. The crystallographic structures of CBD12 variants of NCX1, exhibiting different regulatory properties, show nearly identical interdomain angles between CBD1 and CBD2 and thus, global CBD alignment does not seem to shape regulatory specificities in NCX1 splice variants^6, 19^. In line with this notion, small-angle X-ray scattering (SAXS) studies revealed that Ca^2+^ binding and occlusion at CBD1 of NCX1-CBD12 splice variants induces a shift towards narrowly distributed elongated conformations, as predicted by the population shift mechanism^28^. This population shift of conformational states is essential for regulation, thereby representing a common mechanism for NCX1 splice variants regardless of their regulatory specificity^28^. Finally, hydrogen-deuterium exchange mass-spectrometry (HDX-MS) analyses of NCX1-CBD12 splice variants have shown that Ca^2+^ binding to all tested variants mainly rigidifies the backbone dynamics of CBD2 (and not that of CBD1), where the alternative splicing of CBD2 secondarily modifies the strength and expansion of rigidification throughout CBD2^29, 31^. Importantly, the Ca^2+^-induced effects on CBD2 backbone dynamics correlate well with the regulatory specificity found in the matching splice variants of full-size NCX1s^29, 31^.

Thus far, NCX1 splice variants share a common mechanism for the initial decoding of the regulatory signal upon Ca^2+^ occlusion and tethering of CBDs (i.e., through the population shift mechanism), whereas alternative splicing of CBD2 controls the propagation and strength of Ca^2+^-dependent rigidification and thereby, diversifies the regulatory response^28, 29, 31^. Currently it is unclear to what extent (if at all) the above-described mechanisms are also valid for NCX2 and NCX3 splice variants and how the exons control the CBD dynamics as related to shaping the regulatory specificity in a given isoform/splice variant. To overcome this gap, here we analyzed the apo and Ca^2+^-bound forms of isolated NCX2-CBD12, NCX3-CBD12-AC, and NCX3-CBD12-B proteins with SAXS^19, 28, 29^ and HDX-MS^29, 31^ in order to identify their conformational dynamics and the effect of alternative splicing. The present analysis reveals a general mechanism for regulatory coupling associated with Ca^2+^ interactions with regulatory CBD domains of NCX1, NCX2, and NCX3 isoforms and their splice variants, where the dynamic effects of alternative splicing on conformational dynamics of the two-domain regulatory tandem (CBD12) control the contributions of a given isoform/splice variant to dynamic handling of cytosolic Ca^2+^ oscillations in distinct cell types.

## Results

### Global SAXS parameters of CBD12 proteins

**Figure 2:**
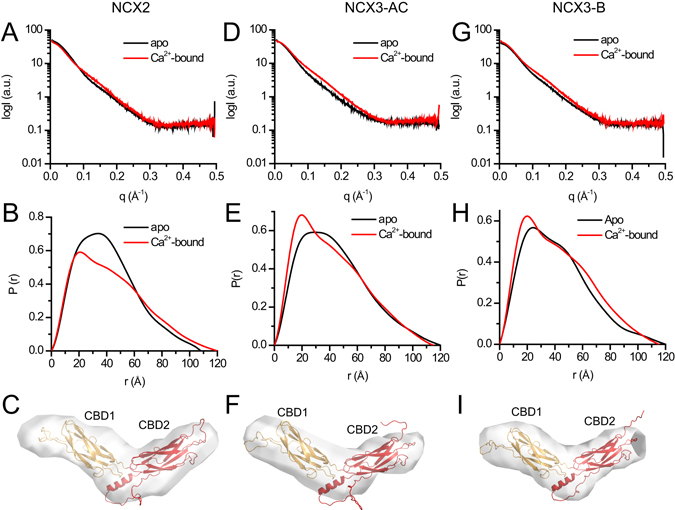
*Ab-initio* modelling of CBD12 from NCX2 and NCX3. (**A**,**D**,**G**) Experimental SAXS curves of CBD12 from NCX2 (**A**), NCX3-AC (**D**), and NCX3-B (**G**) in the presence (red) and absence (black) of Ca^2+^. (**B**,**E**,**H**) Paired-distance distribution functions of CBD12 from NCX2 (**B**), NCX3-AC (**E**), and NCX3-B (**H**) in the presence (red) and absence (black) of Ca^2+^ as determined using GNOM. (**C**,**F**,**I**) *Ab-initio* model of CBD12 from NCX2 (**C**), NCX3-C (**F**), and NCX3-B (**I**) in solution in the presence of Ca^2+^. The homology model of CBD12 was fit into the molecular envelope using SUPCOMB. CBD1 and CBD2 are presented as orange and red cartoons, respectively.

To determine the low-resolution structure-dynamic features of NCX-CBD12 isoforms in solution as well as to resolve the effect of ligand binding on the global conformational states, SAXS was used (Fig. 2, Table 1)^33^. In all the examined proteins (NCX2-CBD12, NCX3-CBD12-AC, and NCX3-CBD12-B), the Ca^2+^-bound form exhibited similar R_g_ and D_max_ values, with PDDF, which represents an elongated conformation (Table 1, Fig. 2B,E,H). The Porod volumes of all the Ca^2+^-bound proteins were similar and matched the volume predicted for a monomeric protein, as previously shown for NCX1-CBD12 (Table 1)^28^. However, in the apo form, the Porod volume matched a volume that is intermediate between a dimer and a monomer (Table 1). Therefore, we pursued further modelling only for the Ca^2+^-bound forms. All three proteins examined here, display similar, elongated molecular envelopes (Fig. 2C,F,I). The CBD12 homology models, based on the crystal structure of NCX1-CBD12-E454K, fit well in the molecular envelopes in all proteins examined here (Fig. 2C,F,I). Taking this into account, along with the low normalized shape discrepancy (NSD) of the models (Table 1) and the features observed in the PDDFs of all three proteins in the Ca^2+^-bound state (Fig. 2B,E,H), it can be assumed that NCX2-CBD12, NCX3-CBD12-AC, and NCX3-CBD12-B exhibit a very similar elongated conformation in a relatively rigid state in solution^33^.

**Table 1:** .

Sample	NCX2-CBD12		NCX2-CBD12-AC		NCX2-CBD12-B	
Ligand	10 mM Ca^2+^	10 mM EDTA	10 mM Ca^2+^	10 mM EDTA	10 mM Ca^2+^	10 mM EDTA
Data collection parameters						
Beamline	ESRF BM29					
Beam geometry (mm^2^)	0.5 × 0.5					
Wavelength (Å)	1.0					
Q range (Å^−1^)	0.0025–0.5					
Exposure time per frame (seconds)^1^	1					
Concentration range (mg/ml)	1.5–10		1.5–11.5		1.5–11.25	
Temperature (°C)	5					
Structural parameters						
R_g_ (Å) [from *P*(*r*)]^2^	34.9 ± 0.01	32.5 ± 0.007	33.9 ± 0.006	35.0 ± 0.01	34.1 ± 0.007	33.5 ± 0.01
R_g_ (Å) (from Guinier)^2^	32.6 ± 0.1	31.5 ± 0.2	32 ± 0.1	36.2 ± 0.4	32.9 ± 0.1	33.5 ± 0.3
D_max_ (Å)^3^	120 ± 12	108 ± 11	115 ± 12	120 ± 12	115 ± 12	120 ± 12
Porod volume [from *P*(*r*)] (10^3^ Å^3^)	45.9	70.0	39.5	72.5	40.5	56.3
NSD^4^	0.85 ± 0.04	NA	0.81 ± 0.05	NA	0.81 ± 0.05	NA
χ^2^ (EOM)	1.5	NA	0.65	NA	0.66	NA
Software employed						
Primary data reduction	AUTOMAR					
Data processing	PRIMUS					
Modelling	DAMMIF, EOM					

^1^Ten frames were measured for each sample. ^2^±S.E. ^3^±10% (estimated range). ^4^NSD, normalized shape discrepancy for DAMMIF calculation.

### Ensemble optimization method (EOM) SAXS analysis of CBD12 proteins

**Figure 3:**
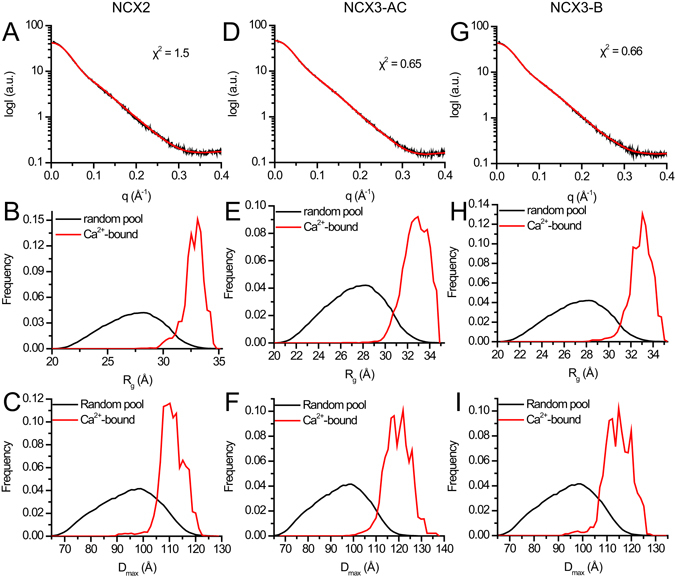
EOM analyses of CBD12 from NCX2 and NCX3. (**A**,**D**,**G**) Experimental SAXS curve and EOM fit of CBD12 from NCX2 (**A**), NCX3-AC (**D**), and NCX3-B (**G**) in the presence of Ca^2+^. (**B**,**E**,**H**) Random R_g_ pool (black line) and EOM-selected ensemble (red line) distributions of NCX2-CBD12 (B), NCX3-CBD12-AC (**E**), and NCX3-CBD12-B (**H**). (**C**,**F**,**I**) Random D_max_ pool (black line) and EOM-selected ensemble (red line) distributions of NCX2-CBD12 (**C**), NCX3-CBD12-AC (**F**), and NCX3-CBD12-B (**I**).

To further assess the conformational distributions of the examined proteins, we performed EOM analysis (Fig. 3). The advantage of EOM analysis is that it can describe the conformational distribution of a molecule instead of representing the molecule using global parameters that do not account for conformational heterogeneity^28, 34^. The selected ensembles fit the experimental data well, as judged by the low χ^2^ values (Table 1). In all three proteins tested here (NCX2-CBD12, NCX3-CBD12-AC, and NCX3-CBD12-B), the conformational distributions are narrow (Fig. 3B,E,H) and biased towards the most elongated conformations (Fig. 3C,F,I), thereby implying a rigid and elongated conformation of Ca^2+^-bound species for all CBD12 variants in solution. Notably, the effect of Ca^2+^ results from binding to CBD1 and not CBD2, since NCX3-CBD12-AC exhibits these properties despite the fact that it does not bind Ca^2+^ at CBD2^26^. These features of NCX2-CBD12, NCX3-CBD12-AC, and NCX3-CBD12-B are somewhat similar to mammalian NCX1-CBD12 splice variants analyzed previously^28^. Taken together, the data supports the notion that Ca^2+^-binding to the primary (high-affinity) sensor of the CBD12 tandem either in NCX1, NCX2, or NCX3 results in a Ca^2+^-induced population shift of preexisting conformational states, where more elongated conformational states become more populated (Fig. 3)^28^.

### Effect of alternative splicing on HDX profiles in NCX3-CBD12

The SAXS analysis, described above, revealed similar behavior in all NCX-CBD12 isoforms and splice variants tested here, exhibiting a rigid and elongated conformation in the presence of Ca^2+^ (Figs 2 and 3). Therefore, it remains unclear how Ca^2+^-dependent NCX regulation is diversified between the different isoforms and splice variants. To better understand the differential conformational dynamics of NCX-CBD12 isoforms and splice variants, the isolated preparations of NCX2-CBD12 and NCX3-CBD12 proteins were analyzed in the apo and Ca^2+^-bound states by HDX-MS^29, 31^.

The identified peptic peptides that were used for HDX-MS analysis are shown in Figure S1. The deuterium uptake levels of each protein in the apo state (Figure S2A,C, and E) or in the Ca^2+^-bound state (Figures S2B,D, and F) are overlaid on the protein structures as color-coded heat maps. It is not feasible to directly compare the deuterium exchange levels between proteins with different sequences because amino acid side chains affect the deuterium uptake rates and different peptic peptides are generated. Therefore, a comparison between NCX2-CBD12 and NCX3-CBD12 variants is not feasible, whereas a comparison between NCX3-CBD12-AC and NCX3-CDB12-B is possible within regions with the same sequences (i.e., outside the alternative splicing regions).

**Figure 4:**
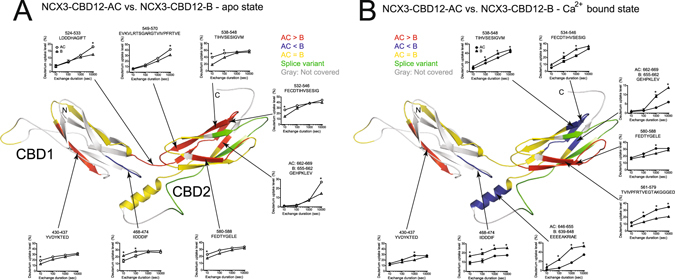
Effect of alternative splicing on the conformational dynamics of NCX3-CBD12. Deuterium uptake differences between NCX3-AC and NCX3-B in the apo form (**A**) or in the Ca^2+^-bound form (**B**) are color-coded onto the model of NCX3-AC. Regions with similar deuterium uptake are yellow, regions with higher deuterium uptake in NCX3-AC are red, and regions with higher deuterium uptake in NCX3-B are blue. The alternative splicing region is in green and regions not covered by the detected peptic peptides are in gray. The uptake plots of the indicated peptic peptides are presented. Error bars represent SEM, * indicated p < 0.05 as analyzed by t-test.

To characterize the effect of alternative splicing on the structural dynamics of NCX3-CBD12, we compared the deuterium exchange levels of NCX3-CBD12-AC and NCX3-CDB12-B using peptic peptides denoted as blue bars in Figure S1B. In both the apo and Ca^2+^-bound states, a few regions of the backbone dynamics considerably differ (Fig. 4) despite the predicted structural similarity and the similar global behavior of the analyzed proteins in solution (Figs 1, 2 and 3). In the apo state, NCX3-CBD12-AC exhibits a higher deuterium uptake than does NCX3-CBD12-B in CBD1, CBD1-CBD2 linker, and CBD2 (Fig. 4A, red regions). On the other hand, the CBD1 EF loop (peptide 468–474), which participates in forming the Ca^2+^-binding sites and interacts with the α-helical region of CBD2^19^, exhibits less uptake in NCX3-CBD12-AC as compared with NCX3-CBD12-B (Fig. 4A, blue region). This result reveals that the alternative splicing segment (Fig. 1, located on CBD2) affects the folding stability of CBD12 in the apo state and thus, may predefine distinct conformational responses of CBDs to Ca^2+^-binding in a given splice variant^6, 29, 31^.

Ca^2+^ binding alters the backbone dynamic differences between the two NCX3-CBD12 variants (Fig. 4B). The deuterium uptake levels within the CBD1-CBD2 linker and nearby regions of CBD2 become similar between NCX3-CBD12-AC and NCX3-CDB12-B (Fig. 4B, yellow regions). The CBD1-CBD2 linker is rigidified to a similar extent in both splice variants (Fig. 4B), consistent with similar conformational distributions observed in the EOM SAXS analysis (Fig. 3); the NCX3-CBD12-AC linker is no longer more dynamic than the NCX3-CBD12-B linker upon Ca^2+^ binding.

The α-helical region of CBD2 [peptides 646–655 (AC) or 639–648 (B)], adjacent to the CBD1 Ca^2+^-binding sites, exhibits markedly lower deuterium uptake in NCX3-CBD12-AC as compared with NCX3-CBD12-B in the Ca^2+^-bound state (Fig. 4B). In addition, the C-terminal tip of CBD2 [peptides 538–548, 534–546, 662–669 (AC) and 655–662 (B)] exhibits a lower deuterium uptake in NCX3-CBD12-AC compared with NCX3-CBD12-B in the Ca^2+^-bound state (Fig. 4B). This finding is especially peculiar in light of a previous analysis demonstrating that NCX3-CBD12-AC lacks the capacity for Ca^2+^ binding at CBD2, whereas NCX3-CBD12-B binds three Ca^2+^ ions at the C-terminal tip of CBD2^30, 34^. Overall, the effect of Ca^2+^ binding on backbone rigidification is more marked in NCX3-CBD12-AC as compared with NCX3-CBD12-B (Fig. 4), which may account for the observed differences in the dissociation kinetics of occluded Ca^2+^ at the interface of CBD1 and CBD2 in NCX3 splice variants^26^.

### HDX profiles of NCX2-CBD12 and NCX3-CBD12

Although a direct comparison of deuterium uptake levels between NCX2-CBD12 and NCX3-CBD12 variants is not feasible, we can compare the regions affected by Ca^2+^ binding.

**Figure 5:**
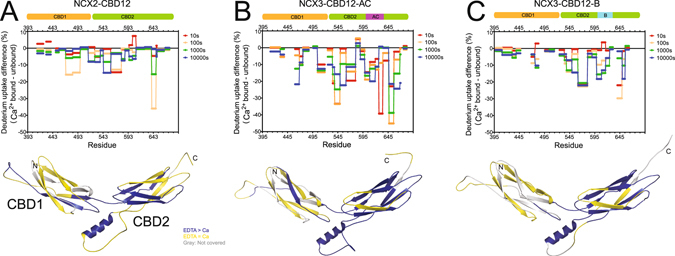
Effect of Ca^2+^ binding on NCX2-CBD12 and NCX3-CBD12 dynamics. Deuterium uptake differences between the Ca^2+^-bound form and the apo form as a function of residue at different time points as indicated are plotted for NCX2-CBD12 (**A**), NCX3-CBD12-AC (**B**), and NCX3-CBD12-B (**C**). The data are also qualitatively depicted on the models of NCX2-CBD12 (**A**), NCX3-CBD12-AC (**B**), and NCX3-CBD12-B (**C**). Regions with lower deuterium uptake in the Ca^2+^-bound form are in blue, regions with similar deuterium uptake in the Ca^2+^-bound form and apo form are in yellow, and regions not covered by the detected peptic peptides are in gray.

To better envisage the Ca^2+^-dependent changes in backbone dynamics, we plotted the difference in deuterium uptake as a function of residue at different deuterium uptake durations, and the Ca^2+^-induced deuterium uptake changes were also color-coded onto the CBD12 model structures (Figs 5 and S3). When examining the effect of Ca^2+^-binding, all three tested proteins exhibit markedly reduced deuterium uptake throughout CBD1 and CBD2 (Figs 5 and S3). This is true even for NCX3-CBD12-AC, which does not bind Ca^2+^ at CBD2^26^. This supports the notion that occupation of the high-affinity primary sensor at CBD1 in NCX2 and NCX3 plays a key role in Ca^2+^-dependent stabilization of CBD2 due to conformational stabilization of the two-domain interface and the interdomain linker upon Ca^2+^ binding and occlusion at the primary sensor at CBD1. A similar structure-dynamic module was suggested for NCX1-CBD12 splice variants based on the HDX-MS analysis^29, 31^.

The interdomain CBD1-CBD2 linker (peptide 513–525 in NCX2 and 524–533 in NCX3) exhibits lower deuterium uptake upon Ca^2+^-binding in all three proteins analyzed here (Figs 5 and S3). This evidence is consistent with the rigid conformations observed in the EOM SAXS analysis (Fig. 2). Similarly to NCX1-CBD12 splice variants^29, 31^, the presently analyzed NCX2 and NCX3 splice variants also exhibit the rigidification of the two-domain interface (including the CBD1-CBD2 linker) upon Ca^2+^ binding, meaning that all NCX1, NCX2, and NCX3 isoform/splice variants, tested until now, share a common mechanism for the initial decoding of regulatory Ca^2+^ signals.

Notably, in all the examined proteins, the α-helical region at the domain’s interface (in the vicinity of the alternative splicing segment, Fig. 1) (peptide 639–647 in NCX2 and 646–655 in NCX3) exhibits lower deuterium uptake in the Ca^2+^-bound state (Figs 5 and S3). This could be important for exon-controlled stabilization of Ca^2+^-dependent CBD tethering, which in turn, affects the strength of Ca^2+^ binding and the off-rates of occluded Ca^2+^ dissociation from CBD1.

## Discussion

Functional contributions of NCX2 and NCX3 variants to physiological and pathological settings have been documented^13, 34^, while underscoring the importance of tissue-specific expression and regulatory diversities exhibited by these isoforms and splice variants^3, 4, 6, 9, 11^. The goal of the present work was to determine the structure-dynamic basis underlying the previously reported regulatory diversities in an isolated CBD12 model and full-size NCX variants in order to formulate the principal mechanism underlying the regulatory diversity in NCX proteins. To this end, the NCX2-CBD12, NCX3-CBD12-AC, and NCX3-CBD12-B proteins were analyzed here by using SAXS and HDX-MS techniques.

As summarized above, the diversity in allosteric regulation of NCX proteins is puzzling at first glance. Some basic mechanisms seem to apply to all isoforms and splice variants, that is, the synergistic interactions between CBD1 and CBD2 are manifested as increased affinity and slower Ca^2+^ dissociation kinetics at CBD1^25, 26^. On the other hand, these interactions are clearly modified by alternative splicing to fulfill specific physiological needs in distinct cell types^25, 26^. In addition, distinct NCX variants exhibit different regulatory responses to Ca^2+^ and Na^+^-dependent regulation despite having high homology and predicted structural similarity^6^. For example, in NCX1, variants that are capable of Ca^2+^ binding at CBD2 exhibit Ca^2+^-dependent alleviation of Na^+^-induced inactivation, whereas in NCX3, NCX3-CBD12-AC, which does not bind Ca^2+^ at CBD2, exhibits less Na^+^-induced inactivation as compared with NCX3-CBD12-B^11, 26^. NCX2 differs from the rest NCX variants in that that it does not undergo alternative splicing and it does own to the Na^+^-induced inactivation. The results presented here, taken together with extensive previous studies of NCX1 splice variants^6, 18, 19, 21, 25, 28, 29, 31, 32^, may provide a conceptual framework to resolve a common mechanistic platform and disparities owned by distinct isoform-splice variants.

First and foremost, CBD1 and CBD2 act as Ca^2+^ sensors. Therefore, their binding affinity and kinetics must correlate with the dynamic properties of Ca^2+^ signaling in a given tissue^1, 2^. In this respect, the importance of alternative splicing is straightforward: NCX1 (NCX1-AD) and NCX3 (NCX3-B, NCX3-BC) splice variants expressed in the brain, as well as NCX2, exhibit faster Ca^2+^ dissociation kinetics from CBD12 compared with NCX1 and NCX3 splice variants expressed in muscles (NCX1-ACDEF in cardiac muscle and NCX3-AC in skeletal muscle)^25, 26^. This is in line with the prolonged Ca^2+^ transients and action potential durations in muscles compared with neurons^4, 5, 26^. These hallmark features, assigned to Ca^2+^ interactions with CBD1 and CBD2, strictly correlate with characteristic regulatory modes ascribed to matching (full-size) NCX variants^4, 7, 9, 11, 13, 25, 26^. The equilibrium and kinetic parameters measured in isolated NCX1-CBD12-ACDEF preparations^25^ were successfully applied to computer-aided dynamic modeling of allosteric activation of the cardiac NCX1 (ACDEF) during the action and resting potentials, thereby providing indispensable information on the dynamic contributions of NCX in handling the Ca^2+^-dependent Ca^2+^-release from the sarcoplasmic reticulum, the Ca^2+^-spark appearance, and duration within local sub-cellular compartments and the fine-tuning of excitation-contraction coupling in cardiomyocytes^35^. Similar applications for computer-aided modeling are straightaway required for clarifying the partial contributions of multiple NCX isoforms and their splice variants to the dynamic handling of dynamic Ca^2+^ swings in neuronal tissues. The emerging working hypothesis is that specific conformational variances, raised by exons, diversify the affinity/off-rates of Ca^2+^ at CBD1 (for controlling the Ca^2+^-dependent allosteric activation) and the affinity/capacity of Ca^2+^ binding to CBD2 (for controlling the Ca^2+^-dependent alleviation of Na^+^-induced inactivation)^25, 26^.

In NCX1, both the brain (AD) and cardiac muscle (ACDEF) splice variants bind Ca^2+^ at CBD2, to alleviate Na^+^-induced inactivation. The ability to counteract Na^+^-induced inactivation is of major importance in excitable tissues where large Na^+^ fluxes (through the voltage-sensitive Na^+^-channels) occur at the initial stage of cell depolarization. NCX2 (expressed in the brain) lacks the Na^+^-induced inactivation, whereas in NCX3 the Na^+^-induced inactivation can be counteracted by increased levels of cytosolic Ca^2+^, but to varying degrees^9, 11^. It was previously proposed that an electrostatic switch at CBD2 underlies the alleviation of Na^+^-induced inactivation upon Ca^2+^ binding, but this proposal cannot account for the regulatory properties of NCX2 (where the Ca^2+^ binding affinity at CBD2 is extremely low) or for regulatory features of NCX3-AC^9, 11, 21, 26^. However, the data presented here may resolve the principal structural platform for the dynamic regulation of NCXs, as well as it can pinpoint the exon-governed fine shaping in the backbone dynamics that may account for regulatory diversity.

In recent years, a growing body of evidence has suggested an updated view of allosteric regulation in proteins. More specifically, a greater importance is given to changes in protein dynamics and conformational distributions rather than to global conformational transitions^36, 37, 38–42^. The accumulating evidence raises several important points. Protein rigidity seems to serve as a common communication route between distant sites in proteins, resulting in structural coupling between the regulatory regions (e.g., CBD12) and the effector site (e.g., the ion-transport domain)^42^. Moreover, in some instances, allosteric interactions are mediated only by dynamic changes, without having any gross effects on the protein conformation^36, 38, 39^. In other cases, the population shift mechanism describes a shift in the protein ensemble of conformations towards active states, where no obvious global conformational change is observed^37^. These observations were recently formulated into a unified model of allostery, based on thermodynamics, the structure, and the free energy landscape of the population shift^41^. According to this general model, the allosteric mechanism in a specific protein requires three elements^41^, allowing us to formulate a general scheme for NCX isoforms and regulation of splice variants.

First, the active conformation responsible for allosteric regulation is required^41^. This conformation is provided by the crystal structure of NCX1-CBD12-AD-E454K^19^, which also allows us to model NCX2-CBD12 and NCX3-CBD12 in the Ca^2+^-bound conformation. The molecular envelopes calculated here (Fig. 2) verify the relevance of these homology models to the global conformations of Ca^2+^-bound CBD12 isoforms. The EOM analyses performed previously for NCX1-CBD12 and performed here for NCX2-CBD12 and NCX3-CBD12 are in line with the unified model, whereby the allosteric event (Ca^2+^ binding) does not create any new conformational states but instead, only shifts the population among the existing states, towards more stable elongated conformations (Fig. 3)^28^.

Second, a set of interacting residues is required^41^. This set of residues is clearly depicted in the crystal structure of NCX1-CBD12-AD-E454K, where a network of hydrogen-bonded salt bridges and hydrophobic interactions between the α-helical region of CBD2 and F450 from NCX1 form the interdomain interface^19^. Notably, the two-domain interface is extremely well conserved among all isoform splice variants, so the use of a crystal structure of NCX1-CBD12-AD-E454K as a universal model is highly justified. According to this model, the mutations of interacting residues must uncouple the regulatory function of CBDs. This prediction has been experimentally demonstrated by showing a lack of Ca^2+^ occlusion and reduced binding affinity of Ca^2+^ in several mutations of residues contributing to the stabilization of the two-domain interface, including the interdomain linker (e.g., G503), the hydrophobic core (e.g., F450), and salt bridge elements (e.g., R532 or D565) tethering CBDs upon Ca^2+^ binding^6, 19, 23–29^. For example, the structural evidence revealed by HDX-MS demonstrates the reduced Ca^2+^-dependent CBD2 rigidification in the F450G mutant, thereby confirming the general model for Ca^2+^-dependent tethering of CBDs and the rigidification of the two-domain interface^19, 29^. Consistent with this statement, this residue is highly conserved among NCX orthologs^19^ and F472 in NCX2-CBD12 and F474 in NCX3-CBD12 (corresponding to F450 in NCX1-CBD12) show reduced deuterium uptake upon Ca^2+^ binding (Figs 4 and 5). Moreover, the accumulating HDX-MS data on NCX1-CBD12, NCX2-CBD12, and NCX3-CBD12 proteins depict the allosteric stabilization within CBD2 upon Ca^2+^ binding at CBD1 (Figs 4 and 5) through the population shift mechanism, shared by all tested isoform/splice variants^6, 28, 29, 31^. Thus, the CBD2 isoforms and splice variants dynamically shape the initial decoding of regulatory signal upon Ca^2+^ binding and thus, the signal message undergoes a transformation toward further propagation of allosteric signal. The underlying mechanisms of signal propagation from the CBDs to the target sites of ion-transport domains remain to be discovered.

Finally, one should consider whether ligand binding at the regulatory site stabilizes the active conformation, destabilizes the inactive conformation, or does both simultaneously^43^. This question is not fully resolved with NCX proteins because a structure of apo-CBD12 is lacking. However, EOM studies of NCX1-CBD12 splice variants show that the active conformations are also sampled in the absence of Ca^2+^, but with a lower frequency^28^. The Ca^2+^-bound crystal structure also demonstrates how the presence of Ca^2+^ “locks” the two domains in an elongated conformation^19^. Thus, the Ca^2+^-dependent stabilization of the one or more “active conformation states” can explain (at least partially) the mechanism by which the ligand binding modulates the activity of NCX proteins.

Having this general mechanism in mind, it can be concluded that different NCX isoforms and splice variants share a similar “active” conformation, which becomes highly populated upon Ca^2+^ binding (Figs 2 and 3)^19, 28, 29^. A very limited number of major “conformational state(s)” display some clear and highly conserved interdomain interactions between residues, although the complete dynamic response in CBD2 is different among distinct isoforms and splice variants^29, 31^. The present (Figs 3, 4 and 5) and previous HDX-MS analyses of the different CBD12 proteins^29, 31^ underscore a general concept according to which the rigidity and allosteric capacity strictly correlate^42^. Intriguingly, the disparate isoforms have different means to control the rigidity of CBD2. That is, in NCX1, alternative splicing using A-exon acts by allowing Ca^2+^ binding at CBD2, which in turn, increases the rigidity of CBD2^25, 29^. In NCX3, the mere presence of exon A results in increased response to regulatory Ca^2+^ binding at CBD1 (Fig. 4). The concept described here is further supported from previous analyses of CBD12 splice variants from CALX, a *Drosophila melanogaster* NCX ortholog, which exhibits anomalous Ca^2+^-dependent regulation. In line with the general mechanism described here, Ca^2+^ binding to CBD1 in CALX1.1 does not rigidify CBD2 and indeed, the intact (full-size) CALX1.1 responds to the regulatory Ca^2+^ binding in a negative mode, thereby exhibiting inhibition rather than activation^29^.

Comparison of HDX-MS profiles of NCX2-CBD12, NCX3-CBD12-AC, and NCX3-CBD12-B (Figs 4, 5 and S2) revealed that in NCX3, the B and AC exons differentially stabilize CBD1 folding in the apo state; thus NCX3-CBD12-B is less dynamic than is NCX3-CBD12-AC. However, these differences are not prominent in the Ca^2+^-bound states, meaning that Ca^2+^ binding results in greater rigidification of NCX3-CBD12-AC and therefore the apo form predefines the incremental effect of Ca^2+^ binding. The greater dynamic effect may explain the reduced Na^+^-dependent inactivation. Similarly, in NCX1-CBD12 proteins, A-exon-containing variants in which Ca^2+^ can alleviate Na^+^-dependent inactivation exhibit greater rigidification of CBD2 (aided by Ca^2+^-binding). Therefore, the observed deviations in the conformational dynamics of the NCX1 and NCX3 splice variants (Fig. 4) adequately match the CBD2-controlled regulatory variances observed in full-size NCX1 and NCX3 splice variants^29, 31^. Although NCX2-CBD12 cannot be directly compared to NCX1-CBD12 and NCX3-CBD12, it also undergoes significant rigidification upon Ca^2+^ binding (Fig. 4).

In general, the Ca^2+^-bound forms of all the tested variants of NCX2-CBD12 and NCX3-CBD12 exhibit more rigid backbone dynamics in comparison with the respective apo-forms (Fig. 5). The Ca^2+^-dependent effects are especially remarkable at the α-helical region of CBD2, which from one side is in proximity with the high-affinity sensor of CBD1 (at the two-domain interface) and on the other side, neighbors the splicing segment at CBD2 (Fig. 1). This mode of Ca^2+^-dependent conformational modulation is consistent with rigidification of the two-domain interface upon Ca^2+^-binding to CBD1, where the tethering of CBDs (through the formation of an interdomain salt-bridge network) stabilizes Ca^2+^ occlusion at high-affinity sites of CBD1, thereby allowing a further transformation and propagation of the allosteric signal. Similar conclusions were drawn for Ca^2+^-dependent rigidification of the α-helix in NCX1-CBD12 variants^29^. Therefore, accumulating evidence strongly supports a general mechanism for the examined NCX1, NCX2, and NCX3 variants, according to which Ca^2+^-binding stabilizes the interdomain linker, the hydrophobic interface between the CBDs, and the nearby α-helix/FG-loop segment on CBD2 next to the splicing segment^9, 25, 28, 29, 31^. It is quite obvious that the Ca^2+^-dependent rigidification of the two-domain interface detected by HDX-MS and SAXS techniques is coupled with the Ca^2+^ occlusion and two-domain tethering of CBDs, which further stabilizes the active conformation in all tested isoform/splice variants of CBD12 proteins^19, 23, 25–32, 44^. The experimental methods described here may be used in future studies to resolve the role of alternative splicing in shaping the allosteric regulation of many protein families.

## Methods

### Overexpression and purification of CBD proteins

The DNA constructs of rat NCX2-CBD12, NCX3-CBD12-AC, and NCX3-CBD12-B were cloned into the pET28a vector, confirmed by sequencing and expressed in *E. coli* Rosetta2 (DE3) competent cells (Novagen) as described^26^. Overexpressed proteins were purified on a TALON-superflow (GE healthcare) column followed by size exclusion chromatography on FPLC by using a Superdex 200 column (GE healthcare)^26^. The purity of the finally purified protein preparations was >95%, as judged by SDS-PAGE. Proteins were concentrated, flash frozen in liquid nitrogen, and stored at −80 °C until their usage for SAXS or HDX-MS experiments.

### Homology modeling of NCX-CBD12 isoforms

The models of NCX2-CBD12, NCX3-CBD12-AC, and NCX3-CBD12-B were generated based on the crystal structure of NCX1-CBD12-E454K (PDB 3US9)^19^. MODELLER was used to generate the models^45^.

### SAXS Data Collection and Analysis

SAXS data were measured at the beamline BM29 of the European Synchrotron Radiation Facility (ESRF), Grenoble, France. Data were collected at 4 °C with the X-ray beam at wavelength λ = 1.0 Å, and the distance from the sample to detector (PILATUS 1 M, Dectris Ltd) was 2.867 meters, covering a scattering vector range (q = 4πsinθ/λ) from 0.0025 to 0.5 Å^−1^. Ten frames of two-dimensional images were recorded for each buffer or sample, with an exposure time of 2 sec per frame. The 2D images were reduced to one-dimensional scattering profiles and the scattering of the buffer was subtracted from the sample profile using the software at the site. The buffers contained 100 mM KCl, 20 mM β-mercaptoethanol, 20 mM Tris-HCl at pH 7.2, and either 10 mM CaCl_2_ or 10 mM EDTA. To account for possible inter-particle effects, each sample was measured at four concentrations: ~10, ~5, ~2.5, and ~1.5 mg/mL. In the presence of such effects, the lowest concentration curve was merged with a higher concentration curve at q ~ 0.2 Å^−1^ to prevent distortion of the low-angle data while preserving a high signal-to-noise ratio at the higher angles, which are far less sensitive to interparticle effects^33^. The experimental radius of gyration (*R*_g_) was calculated from data at low *q* values in the range of *qR*_g_ < 1.3, according to the Guinier approximation: ln*I*(*q*) ≈ ln(*I*(0)) − *R*_g_^2^ *q* ^2^/3 using PRIMUS^46^. The D_max_ values and the Porod volume were derived from the paired-distance distribution function (PDDF or *P(r)*) calculated using GNOM^47^. Ab initio shape reconstructions were performed with the ATSAS software suite, using scattering data of the q range between 0.02 and 0.30 Å^−1^ ^47^.

### Ensemble optimization method (EOM) analysis

NCX2-CBD12, NCX3-CBD12-AC, and NCX3-CBD12-B were modelled with MODELLER^45^, based on the crystal structure of NCX1-CBD12-E454K (PDB 3US9)^19^. Unstructured residues at the N- and C-termini of CBD1 and CBD2 and the large flexible FG-loop of CBD2 were truncated. The software RANCH^48^ was used to generate a pool of 10,000 stereochemically feasible structures with a random CBD1-CBD2 linker, N- and C-termini, and a CBD2 FG-loop. These pools were used as input for GAJOE^48^, which selects an ensemble with the best fit to the experimental data using a genetic algorithm: 50 ensembles of 20 orientations each were “crossed” and “mutated” for 1,000 generations and the process was repeated 50 times.

### Deuterium exchange for HDX-MS experiments

Purified proteins were prepared at a concentration of 100 µM in a buffer composed of 20 mM Tris-HCl, pH 7.2, 100 mM KCl, 20 mM β-mercaptoethanol either with 1.42 mM EDTA or 10 mM CaCl_2._ The HDX reaction was initiated by mixing 2ul of protein samples with 28 µL of D_2_O buffer (20 mM Tris-HCl, pD 7.2, 100 mM KCl, 20 mM β-mercaptoethanol) containing 1.42 mM EDTA, or 10 mM CaCl_2_ for 10 sec, 100 sec, 1,000 sec, or 10,000 sec at 4 °C. The reaction was stopped by adding 30 µl of ice-cold quench buffer (0.1 M NaH_2_PO_4_, 1 M guanidine, 20 mM TCEP, pH 2.01). For non-deuterated (ND) samples, 2 µl of protein samples were mixed with 28 µl of H_2_O buffer (20 mM Tris-HCl, pH 7.2, 100 mM KCl, 20 mM β-mercaptoethanol), and 30 µl of ice-cold quench buffer was added.

### Mass spectrometry, peptide identification, and HDX-MS data processing

The quenched samples were digested and analyzed by the HDX-UPLC-ESI-MS system (Waters) as previously described^29^. Mass-spectral analyses were performed with a Xevo G2 Quadruple-time of fly (Q-TOF) equipped with a standard electrospray ionization (ESI) source in MS^E^ mode in positive ion mode. All setting conditions for the system were reported previously^49^. Identification and HDX-MS data processing of peptic peptides were performed by ProteinLynx Global Server 2.4 (Waters) and DynamX 2.0 (Waters) as previously described^31^. All of the data were derived from at least three independent experiments, and the statistical significance was analyzed by t-test.

## Electronic supplementary material

Supplementary Information
