# Supplementary material for: Structure-based dynamic arrays in regulatory domains of sodium-calcium exchanger (NCX) isoforms
Source: Sci Rep. 2017 Apr 20;7:993. doi: 10.1038/s41598-017-01102-x (PMC5430519; doi:10.1038/s41598-017-01102-x)

Supplementary information

Structure-based dynamic arrays in regulatory domains of sodium-calcium exchanger (NCX) isoforms

Moshe Giladi[1,#], Su Youn Lee [2,#], Yarden Ariely[1], Yotam Teldan[1], Rotem Granit[1],

Roi Strulovich[1], Yoni Haitin[1], Ka Young Chung[2,*], and Daniel Khananshvili[1,*]

[1]Department of Physiology and Pharmacology, Tel-Aviv University, Tel-Aviv 69978, Israel

[2]School of Pharmacy, Sungkyunkwan University, Jangan-gu, Suwon, 440-746, South Korea

[#] These authors contributed equally to this work

[*]Corresponding authors:

Professor Ka Young Chung,
School of Pharmacy, Sungkyunkwan University,
Jangan-gu, Suwon, 16419, South Korea,
E-mail kychung2@skku.edu

Professor Daniel Khananshvili
Department of Physiology and Pharmacology
Sackler School of Medicine, Tel-Aviv University
Ramat-Aviv, Tel-Aviv 69978, Israel
Tel: 972-3-640-9961; Fax: 972-3-640-9113
E-mail: dhanan@post.tau.ac.il

Running title: Isoform-specific regulatory modes in NCX2 and NCX3

Supplementary figure legends

Figure S1. Sequence coverage of NCX2-CBD12 and NCX3-CBD12. (A) Peptic peptides of NCX2-CBD12 are displayed as blue bars. (B) Peptic peptides of NCX3-CBD12-AC are displayed as pink bars, peptic peptides of NCX3-CBD12-B are displayed as light-blue bars, and peptic peptides identical in both splice variants are displayed as blue bars.

Figure S2. HDX-MS profiles of NCX2-CBD12 and NCX3-CBD12. HDX-MS profiles of apo NCX2-CBD12 (A), Ca^{2+}-bound NCX2-CBD12 (B), apo NCX3-CBD12-AC (C), Ca^{2+}-bound NCX3-CBD12-AC (D), apo NCX3-CBD12-B (E), and Ca^{2+}-bound NCX3-CBD12-B (F) at different deuterium uptake duration (as indicated) are overlaid on the models of the respective proteins.

Figure S3. HDX-MS uptake plots of NCX2-CBD12 and NCX3-CBD12. The uptake plots of selected peptides affected by Ca^{2+} binding in NCX2 (A), NCX3-AC (B), and NCX3-B (C) are displayed. * $p < 0.05$.

Figure S1

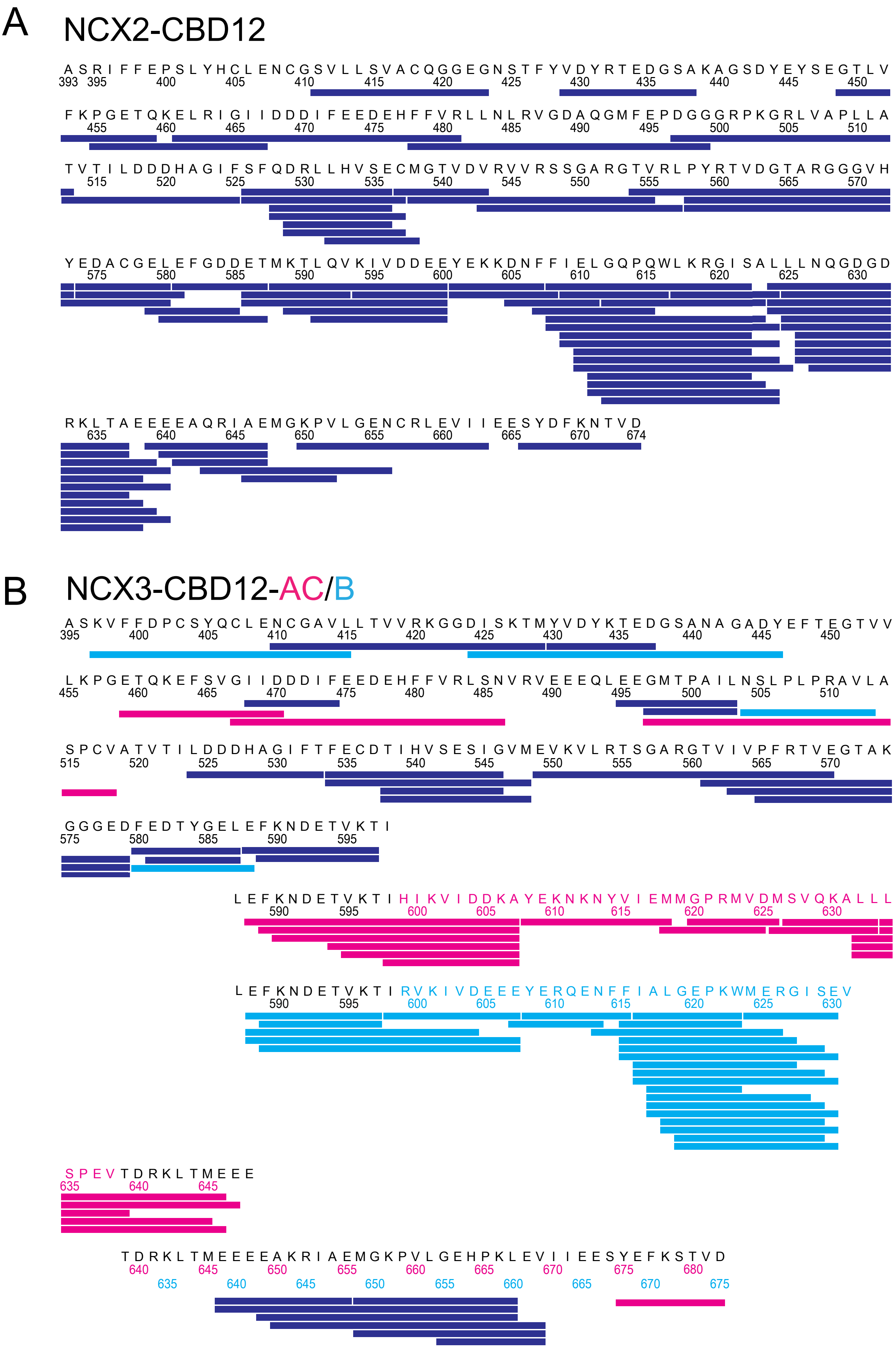
A
NCX2-CBD12
A S R I F F E P S L Y H C L E N C G S V L L S V A C Q G G E G N S T F Y V D Y R T E D G S A K A G S D Y E Y S E G T L V
F K P G E T Q K E L R I G I I D D D I F E E D E H F F V R L L N L R V G D A Q G M F E P D G G G R P K G R L V A P L L A
T V T I L D D D H A G I F S F Q D R L L H V S E C M G T V D V R V V R S S G A R G T V R L P Y R T V D G T A R G G G V H
Y E D A C G E L E F G D D E T M K T L Q V K I V D D E E Y E K K D N F F I E L G Q P Q W L K R G I S A L L L N Q G D G D
R K L T A E E E E A Q R I A E M G K P V L G E N C R L E V I I E E S Y D F K N T V D
B
NCX3-CBD12-AC/B
A S K V F F D P C S Y Q C L E N C G A V L L T V V R K G G D I S K T M Y V D Y K T E D G S A N A G A D Y E F T E G T V V
L K P G E T Q K E F S V G I I D D D I F E E D E H F F V R L S N V R V E E E Q L E E G M T P A I L N S L P L P R A V L A
S P C V A T V T I L D D D H A G I F T F E C D T I H V S E S I G V M E V K V L R T S G A R G T V I V P F R T V E G T A K
G G G E D F E D T Y G E L E F K N D E T V K T I
L E F K N D E T V K T I H I K V I D D K A Y E K N K N Y V I E M M G P R M V D M S V Q K A L L L
L E F K N D E T V K T I R V K I V D E E E Y E R Q E N F F I A L G E P K W M E R G I S E V
S P E V T D R K L T M E E E
T D R K L T M E E E E A K R I A E M G K P V L G E H P K L E V I I E E S Y E F K S T V D

Figure S2

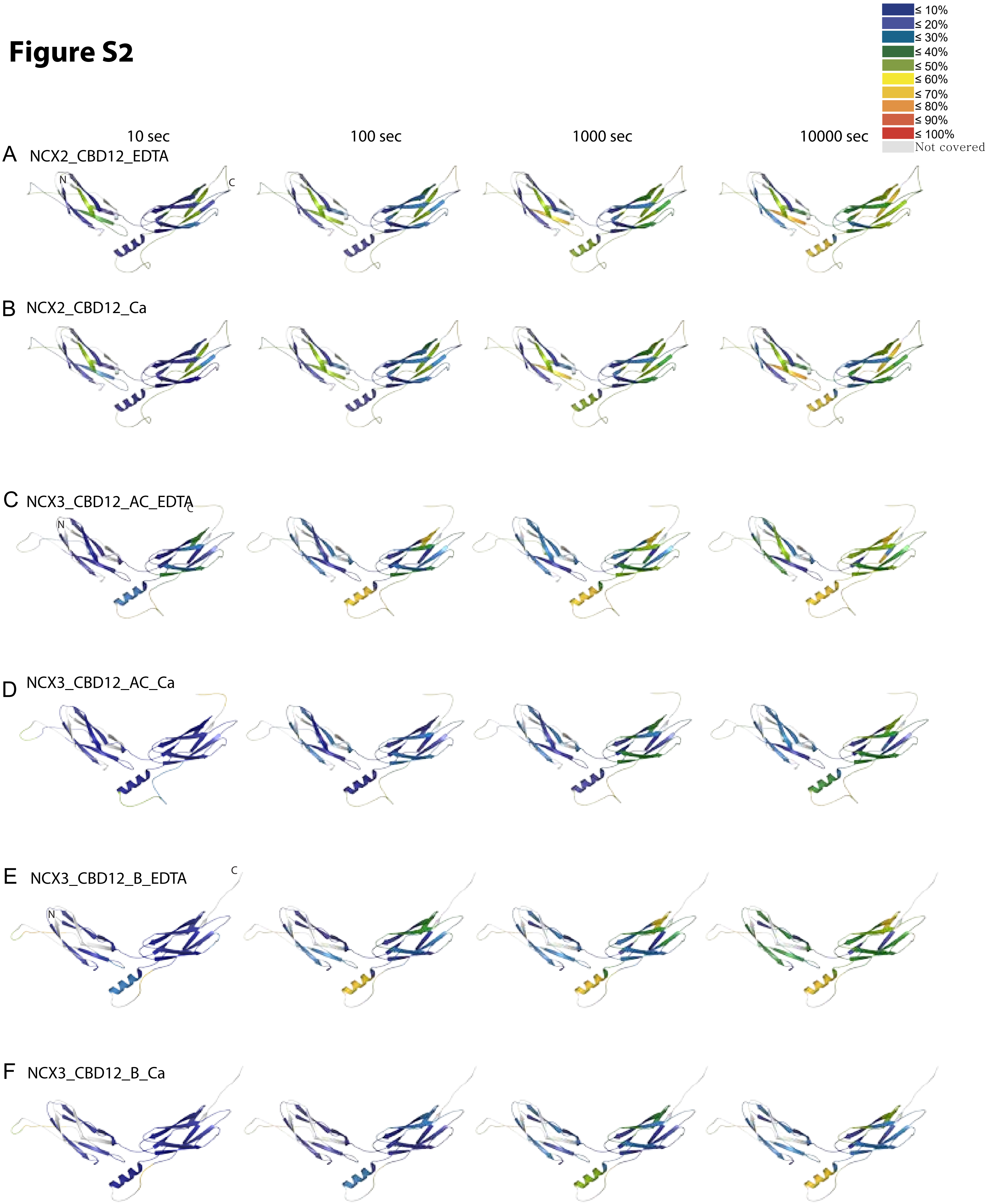
≤ 10%
≤ 20%
≤ 30%
≤ 40%
≤ 50%
≤ 60%
≤ 70%
≤ 80%
≤ 90%
≤ 100%
Not covered
10 sec
100 sec
1000 sec
10000 sec
A NCX2_CBD12_EDTA
N
C
B NCX2_CBD12_Ca
C NCX3_CBD12_AC_EDTA
N
C
D NCX3_CBD12_AC_Ca
E NCX3_CBD12_B_EDTA
C
N
F NCX3_CBD12_B_Ca

NCX2_CBD12

Figure S3

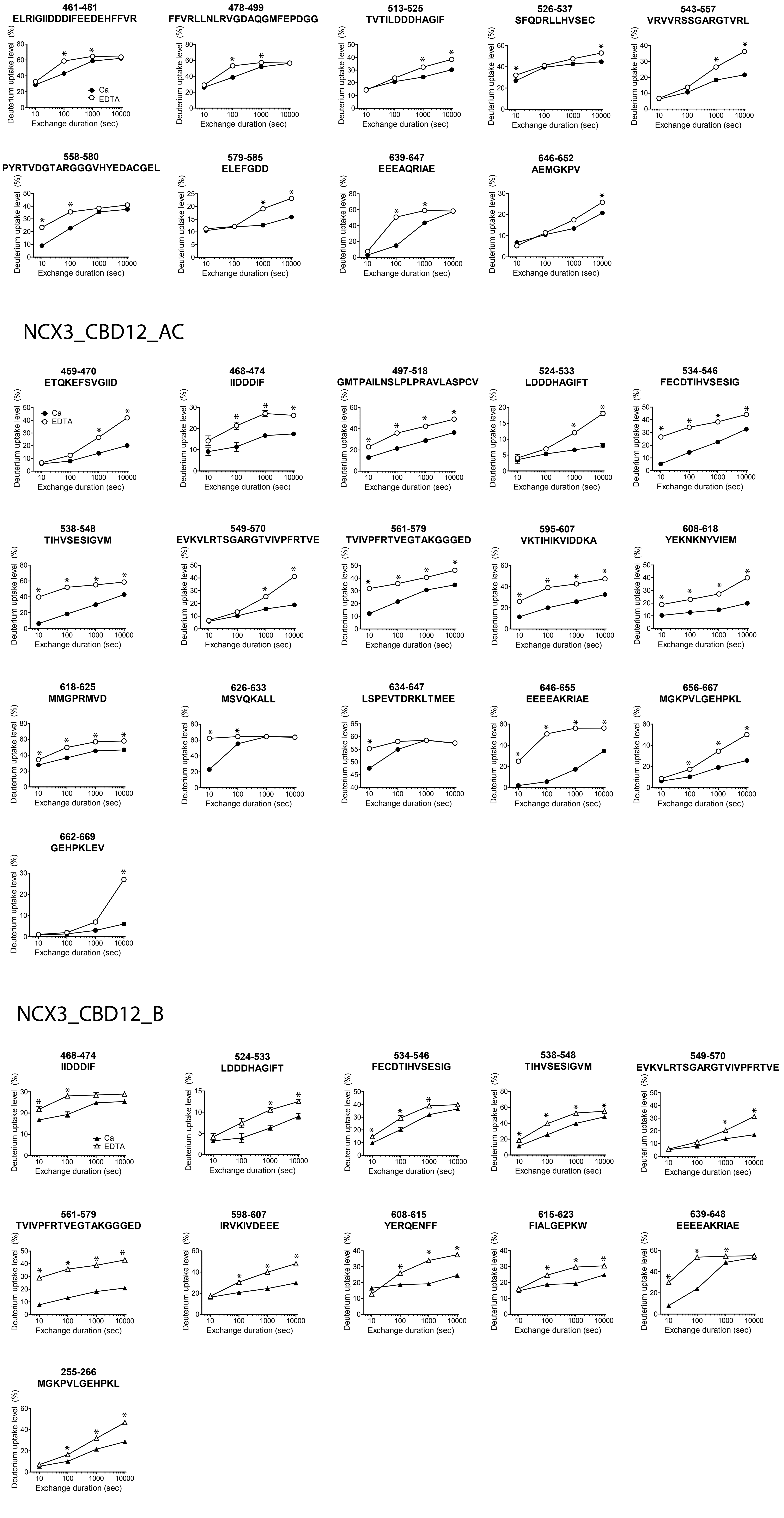

Supplement: Supplementary file 1 — Supplementary Information [file 41598_2017_1102_MOESM1_ESM.pdf]
